# Gastric epithelial dysplasia: characteristics and long-term follow-up results after endoscopic resection according to morphological categorization

**DOI:** 10.1186/s12876-015-0249-7

**Published:** 2015-02-12

**Authors:** Dong Hoon Baek, Gwang Ha Kim, Do Youn Park, Bong Eun Lee, Hye Kyung Jeon, Won Lim, Geun Am Song

**Affiliations:** 1 Department of Internal Medicine, Pusan National University School of Medicine, and Biomedical Research Institute, Pusan National University Hospital, 179, Gudeok-ro, Seo-gu, Busan, 602-739 Korea; 2 Department of Pathology, Pusan National University School of Medicine, and Biomedical Research Institute, Pusan National University Hospital, Busan, Korea

**Keywords:** Stomach, Gastric epithelial dysplasia, Endoscopic resection

## Abstract

**Background:**

Gastric epithelial dysplasia (GED) can be morphologically categorized into adenomatous and foveolar types. To date, there have been few studies on the clinical characteristics of GEDs according to the morphologic types. Therefore, we here aimed to elucidate the clinicopathologic characteristics of patients with GED and the long-term follow-up results after endoscopic resection according to the morphologic characteristics of GEDs.

**Methods:**

A total of 357 patients who underwent endoscopic resection for GEDs at Pusan National University Hospital between January 2008 and December 2009 were included in the study. GEDs were morphologically categorized into adenomatous, foveolar, and hybrid types on histologic examination. The clinicopathologic characteristics of patients with GEDs and outcomes of endoscopic resection were analyzed.

**Results:**

Patients with GED were divided into 3 groups: adenomatous (n = 167, 46.8%), foveolar (n = 103, 28.9%), and hybrid (n = 87, 24.3%) types. Compared to the adenomatous type, foveolar type lesions were more frequently located in the antrum/pylorus, flat/depressed lesions, and normal/reddish in color; and showed more frequent high-grade dysplasia. During the follow–up period (median, 37.3 months), the overall incidence of synchronous and metachronous lesions was 20.8% and 20.1%, respectively; of these, the incidence of synchronous and metachronous gastric cancer was 8.7% and 5.4%, respectively. There were no significant differences in the incidence of synchronous and metachronous lesions according to morphologic types.

**Conclusion:**

GEDs appear to have different clinicopathologic characteristics according to morphologic types. Irrespective of the morphology, synchronous and metachronous gastric cancers are commonly found after endoscopic resection of GEDs. Therefore, close follow-up surveillance after endoscopic resection of GEDs should be performed for all patients.

## Background

Gastric epithelial dysplasia (GED) is an unequivocal neoplastic non-invasive proliferation widely accepted as a precursor to gastric adenocarcinomas [1]. The frequency of GED markedly increases with age, especially in patients in their fifth decade of life and above. This tendency may be related to atrophic changes, and especially intestinal metaplasia of the gastric mucosa, among elderly people [2-4]. The prevalence of GED shows considerable geographic differences, with rates between 0.5% and 3.8% observed in western countries, as compared in to between 9% and 20% in regions with a high prevalence of gastric cancer [5-7]. In addition, the prevalence of GED has been clearly shown to be associated with the regional prevalence of *Helicobacter pylori* infection [8].

GED, which encompasses gastric adenoma, is a relatively common disease entity in Korea. GED lies histologically and clinically on the borderline of benign and malignant lesions, and its natural history is still unclear. Therefore, there is currently no specific treatment policy for GEDs, and the treatments vary from close endoscopic follow-up to endoscopic resection. However, GEDs have been previously demonstrated to represent a penultimate state of gastric carcinogenesis, and to be indicators of an increased risk of synchronous adenocarcinoma elsewhere in the stomach [9-11]. On the basis of these data, endoscopic resection has been recently recommended as the standard treatment for GEDs after prior histologic confirmation of dysplasia [12-14].

GED has traditionally been categorized into adenomatous (intestinal/type I) and foveolar (gastric/type II) types, on the basis of its morphologic characteristics [11,15,16]. Although prior studies have suggested that the foveolar type is almost always low-grade [17,18], the findings of our previous study indicated that foveolar type lesions are more frequently high-grade when evaluated in a high-risk population [19-21], and other studies have indicated that the foveolar type is more commonly associated with poorly-differentiated adenocarcinoma [3,22,23].

However, to date, there have been few studies on the clinical characteristics of the 2 morphologically well-recognized types of GED [15-19], especially in terms of the long-term outcomes after endoscopic resection. Thus we here aimed to elucidate the clinicopathologic characteristics (including endoscopic findings) of each type in patients having undergone endoscopic resection for GED, and to investigate their long-term outcomes after endoscopic resection.

## Methods

**Figure 1:**
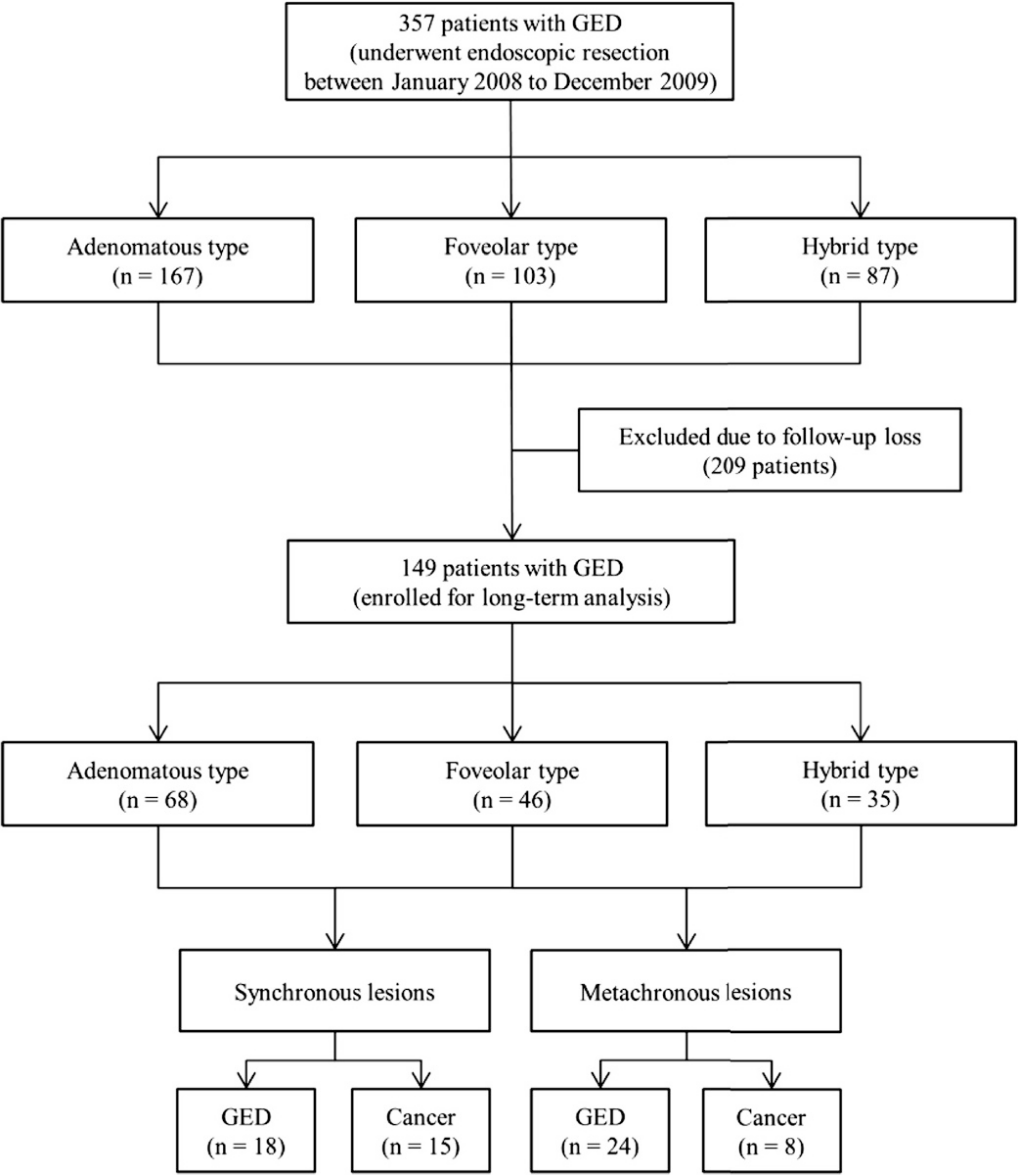
**Flow chart of the patient inclusion in the study.** GED, gastric epithelial dysplasia.

We retrospectively collected and evaluated data of endoscopic submucosal dissection (ESD) and endoscopic mucosal resection (EMR) from our endoscopic database system. From January 2008 to December 2009, 357 patients diagnosed with GED were treated by endoscopic resection (ESD or EMR) at Pusan National University Hospital, Busan, Korea. GEDs were morphologically categorized into adenomatous, foveolar, and hybrid types on histologic examination. The patient characteristics (age, sex, and *H. pylori* infection status), endoscopic findings (location, macroscopic shape, and color of GEDs), and histopathologic features (tumor size, presence of ulceration, and histologic grade) were examined according to the morphological categorization of GEDs. Subsequently, the short-term outcomes, such as en bloc and complete resection rates; and the long-term outcomes, such as detection of synchronous and metachronous lesions, including GED or early gastric cancer (EGC), were analyzed (Figure 1).

The study protocol was reviewed and approved by the Institutional Review Board at Pusan National University Hospital (E-2013007), and written informed consent for the endoscopic resection was obtained from all patients before the procedure.

### Endoscopic findings of GED

Locations of the GEDs were classified as longitudinal (body/fundus or antrum/pylorus) and circular directions (lesser curvature, greater curvature, anterior wall or posterior wall). The macroscopic shapes of the lesions were categorized as protruding (I), non-protruding and non-excavated (II), or excavated (III). Type II lesions were subclassified as slightly elevated (IIa), flat (IIb), or slightly depressed (IIc) [24]. Next, all lesions were broadly classified into 2 groups: protruded/elevated (I, IIa) and flat/depressed (IIb, IIc, III) types. The colors of the lesions were categorized as being either discolored or normal/reddish. Discolored lesions were defined as endoscopically pale in color as compared with the surrounding non-neoplastic mucosa, as opposed to normal/reddish lesions, which were defined as endoscopically reddish or similar in color to the surrounding mucosa.

### Histopathologic evaluation

**Figure 2:**
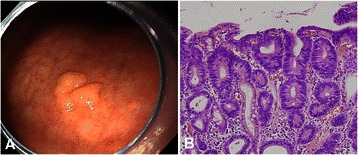
**Representative endoscopic and histologic findings of an adenomatous-type gastric epithelial dysplasia. (A)** An elevated lesion with nodular changes is seen at the lesser curvature of the lower body. **(B)** On histology, tubules lined by columnar cells with hyperchromatic, pencillate nuclei with pseudostratification, and little branching or irregularity are noted (hematoxylin and eosin stain, ×200).

**Figure 3:**
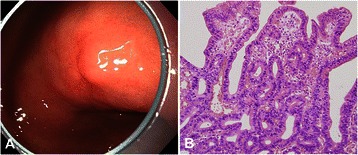
**Representative endoscopic and histologic findings of a foveolar-type gastric epithelial dysplasia. (A)** A slightly depressed lesion is seen at the lesser curvature of the antrum. **(B)** On histology, cuboidal to columnar cells with pale cytoplasm and basally located ovoid nuclei with branching, budding and a cribriform pattern are observed (hematoxylin and eosin stain, ×200).

**Figure 4:**
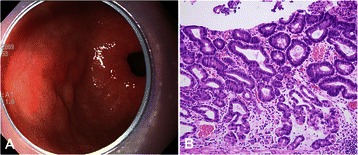
**Representative endoscopic and histologic findings of a hybrid-type gastric epithelial dysplasia. (A)** A nodular lesion with slight central depression is seen at the anterior wall of the antrum. **(B)** On histology, features of both foveolar-type and adenomatous-type dysplasias are observed (hematoxylin and eosin stain, ×200).

All resected specimens were examined by the same expert pathologist (D.Y. Park). All specimens were routinely fixed in 10% buffered formalin, serially sectioned, embedded in paraffin, cut into 2-mm sections, and stained with hematoxylin and eosin as per standard protocol. Each lesion was classified as being either adenomatous, foveolar, or hybrid type according to its morphologic features, as previously described [16,19]. Briefly, adenomatous GEDs resemble colonic adenomas and are composed of large tubules lined by basophilic columnar cells with hyperchromatic pencillate nuclei with pseudostratification, and dense eosinophilic cytoplasm. Goblet and Paneth cells are commonly seen in this form of GED (Figure 2). Conversely, foveolar GEDs show cuboidal to columnar cells with pale-to-clear cytoplasm and hyperchromatic round-to-oval nuclei. Hyperplasia of the foveolar region with irregular glandular branching and epithelial folding is also frequently noted in the foveolar type, whereas Goblet and Paneth cells are rarely identified (Figure 3). Cases of GED showing at least 10% of a second phenotype were classified as hybrid type (Figure 4). Each case was also graded as either low- or high-grade according to previously defined and established criteria, including the presence of architectural complexity and cytologic atypia [16,25]. Presence of ulceration was defined as rupture of the muscularis mucosae or fibrosis in the submucosal layer within the GEDs. En bloc resection was defined as resection in a single piece as opposed to piecemeal resection (multiple segments). Complete resection was defined as successful en bloc resection, with lateral and vertical margins histologically free of neoplasm.

### Follow-up after endoscopic resection

Patient follow-up was based on only endoscopy. The starting date of the follow-up was defined as the date of endoscopic resection (ESD or EMR), and the end of the follow-up was the last date of follow-up endoscopy. Follow-up endoscopy was performed at 6 months after ESD or EMR, and annually thereafter. Biopsy was performed at sites suspicious of harboring synchronous or metachronous lesions. A synchronous lesion, including GED and EGC, was defined as either a concomitant lesion at the time of ESD/EMR, or a lesion detected within a 12-month period after ESD or EMR [26,27]. For patients with initial synchronous lesions, the most dysplastic lesion was considered the main lesion. Similarly in cases of initially detected multiple lesions with the same histology, the largest lesion was considered the main lesion. A metachronous lesion, including GED and EGC, was defined as a lesion diagnosed 12 months after ESD or EMR for the primary lesion, and located in a different part of the stomach, so as not to represent recurrence [26,27]. Local recurrence was defined as GED detected at the site of an endoscopic resection scar.

### Statistical analysis

Quantitative data (age and tumor size) were expressed by mean and standard deviation (SD). Differences in clinicopathologic features among the 3 GED types were evaluated by using the one-way analysis of variance followed by Tukey’s post-hoc test for continuous variables, and the χ2 test or Fisher’s exact test for categorical variables. Short-term outcomes (en bloc and complete resection rates) and long-term outcomes (incidence of synchronous and metachronous lesions) after endoscopic resection according to the GED type and histologic grade were analyzed by using the χ2 test or Fisher’s exact test. A *p*-value of < 0.05 was considered statistically significant. Statistical calculations were performed using SPSS version 17.0 software for Windows (SPSS, Chicago, IL, USA).

## Results

### Clinicopathologic characteristics of the GED patients

The clinicopathologic characteristics of the enrolled 357 patients are summarized in Table 1. The patients included 246 men and 111 women, with a mean age of 62.8 years (range, 36–85 years). On the basis of the morphological features of GED on histologic examination, the patients were divided into 3 groups: adenomatous (n = 167), foveolar (n = 103), and hybrid type (n = 87). There was no history of familial polyposis syndrome in any patient. All 3 types of GED occurred more commonly in men than in women, and there were no statistical significant differences in terms of patient age, gender, and prevalence of *H. pylori* infection among the 3 morphologic groups of GED (*p* = 0.732, *p* = 0.907, and *p* = 0.284, respectively).

**Table 1:** **Clinicopathologic characteristics of patients with gastric epithelial dysplasia according to morphological types**

	Morphologic types			*p*-value
	Adenomatous	Foveolar	Hybrid	
	(n = 167)	(n = 103)	(n = 87)	
Age (years, mean ± SD)	64.0 ± 9.2	60.8 ± 9.0	62.9 ± 9.3	0.732
Gender, male:female	117:50	70:33	59:28	0.907
*H. pylori* infection	134 (80.2)	77 (74.8)	73 (84.1)	0.284
Location (longitudinal)				<0.001
Body/fundus	98 (58.7)	26 (25.2)	26 (29.9)	
Antrum/pylorus	69 (41.3)	77 (74.8)	61 (70.1)	
Location (circular)				0.023
LC	79 (47.3)	32 (31.1)	32 (36.8)	
GC/AW/PW	88 (52.7)	71 (68.9)	55 (63.2)	
Macroscopic shape				< 0.001
Elevated/protruded	101 (60.5)	39 (37.9)	33 (37.9)	
Flat/depressed	66 (39.5)	64 (62.1)	54 (62.1)	
Color				< 0.001
Discolored	118 (70.1)	22 (21.4)	33 (37.9)	
Normal/reddish	49 (29.9)	81 (78.6)	54 (62.1)	
Ulceration	4 (2.4)	7 (6.8)	4 (4.6)	0.211
Size (cm, mean ± SD)	1.5 ± 0.9	1.2 ± 0.7	1.8 ± 2.5	0.093
Histologic grade				0.002
Low	127 (76.0)	66 (64.1)	48 (55.2)	
High	40 (24.0)	37 (35.9)	39 (44.8)	

Values are expressed as n (%).

LC, lesser curvature; GC, greater curvature; AW, anterior wall; PW, posterior wall.

When the locations of the GEDs were divided according to the longitudinal and circular directions, adenomatous type lesions were found to be more frequently located in the body/fundus and in the lesser curvature side than the foveolar and hybrid types (58.7% vs. 25.2% and 29.9%, *p* < 0.001; 47.3% vs. 31.1% and 36.8%, *p* = 0.023, respectively). On macroscopic examination of the GEDs, a flat/depressed shape was more commonly observed in the foveolar and hybrid types than in the adenomatous type (62.1% and 62.1% vs. 39.5%, *p* < 0.001), whereas discoloration was observed more frequently in the adenomatous type than in the foveolar and hybrid types (70.1% vs. 21.4% and 37.9%, *p* < 0.001).

The mean size of the foveolar type lesions was smaller than that of the adenomatous and hybrid types (1.2 cm vs. 1.5 cm and 1.8 cm, respectively), although this was not statistically significant (*p* = 0.093). Ulceration was rare in all 3 types (*p* = 0.211). In terms of the histologic grade, the foveolar and hybrid types showed high-grade histology significantly more frequently than adenomatous type lesions (35.9% and 44.8% vs. 24.0%, *p* = 0.002).

### Short-term outcomes of patients with GED having undergone endoscopic resection

ESD and EMR were performed in 21 (5.9%) and 336 (94.1%) lesions, respectively (Table 2). The en bloc and piecemeal resection rates were 97.8% (349/357), and 2.2% (8/357), respectively. Of the 349 en bloc-resected lesions, 51 lesions were found to be incomplete resections, owing to lateral involvement of the tumor cells in 46 cases, and impossible margin assessment caused by the cauterization artifact in 5 cases. Accordingly, the complete resection rate was 83.5% (298/357). Interestingly, the complete resection rate in adenomatous type lesions was significant lower than that in the foveolar and hybrid types (78.4% vs. 84.5% and 92.0%, *p* = 0.022).

**Table 2:** **Short-term outcomes of patients with gastric epithelial dysplasia having undergone endoscopic resection**

	Morphologic types			*p*-value
	Adenomatous	Foveolar	Hybrid	
	(n = 167)	(n = 103)	(n = 87)	
En bloc resection				
EMR	7/7 (100)	11/11 (100)	3/3 (100)	1.000
ESD	155/160 (96.9)	89/92 (96.7)	84/84 (100)	0.255
Total	162/167 (97.0)	100/103 (97.1)	87/87 (100)	0.267
Complete resection				
EMR	4/7 (57.1)	9/11 (81.8)	3/3 (100)	0.282
ESD	127/160 (79.4)	78/92 (84.8)	77/84 (91.7)	0.044
Total	131/167 (78.4)	87/103 (84.5)	80/87 (92.0)	0.022

Values are expressed as n (%).

EMR, endoscopic mucosal resection; ESD, endoscopic submucosal dissection.

### Long-term outcomes of patients with GED having undergone endoscopic resection

Of the 357 patients, we excluded 208 patients who were followed for <1 year, resulting in 149 patients who underwent endoscopic resection for GED being included in our long-term outcome analysis (Table 3). Synchronous lesions were observed in 31 patients (20.8%) during the study period (18 GEDs and 13 EGCs). There was no statistically significant difference in the incidence of synchronous lesions according to the morphological types (*p* = 0.088). In all 18 patients with synchronous GEDs, complete resection was achieved by ESD. Moreover, all 13 patients with EGC underwent ESD, and all resected carcinomas were differentiated-type adenocarcinomas limited to the mucosa without lymphovascular involvement: 12 (92.3%) were well-differentiated tubular adenocarcinomas and 1 (7.7%) were moderately differentiated. However, one patient underwent additional gastrectomy because of incomplete resection after ESD.

**Table 3:** **Long-term outcomes of patients with gastric epithelial dysplasia having undergone endoscopic resection**

	Morphologic type			*p*-value
	Adenomatous	Foveolar	Hybrid	
	(n = 68)	(n = 46)	(n = 35)	
Synchronous lesions	19 (27.9)	5 (10.9)	7 (20)	0.088
GED	11 (16.2)	4 (8.7)	3 (8.6)	0.372
Cancer	8 (11.8)	1 (2.2)	4 (11.4)	0.166
Metachronous lesions	13 (19.1)	9 (19.6)	8 (22.9)	0.898
GED	10 (14.7)	5 (10.9)	7 (20)	0.518
Cancer	3 (4.4)	4 (8.7)	1 (2.9)	0.459

Values are expressed as n (%).

GED, gastric epithelial dysplasia.

The overall incidence of metachronous lesions after endoscopic treatment was 20.1% (30/149) during a median follow-up period of 37.3 months (range, 12–70 months); of these, the incidence of metachronous GED and carcinoma was 14.8% (22/149) and 5.4% (8/149), respectively. There was no statistically significant difference in the incidence of metachronous lesions according to the morphological types (*p* = 0.898). The median interval from endoscopic resection of GED to detection of the first metachronous neoplasm was 34 months (range, 15–69 months). In all 22 patients with metachronous GEDs, complete resection was achieved by ESD. In the 8 patients with metachronous carcinoma, 7 patients underwent a second ESD, and all resected carcinomas were found to be differentiated-type adenocarcinomas limited to the mucosa without lymphovascular involvement: 4 (57.1%) were well-differentiated tubular adenocarcinomas and 3 (42.9%) were moderately differentiated. One patient with carcinoma underwent surgery because of poorly differentiated histology (signet ring cell carcinoma). In addition, there were no statistical significant differences in the incidence of synchronous and metachronous lesions according to the histologic grading of GEDs high-grade vs. low-grade, 16.0% vs. 23.2%, *p* = 0.311; 24.0% vs. 18.2%, *p* = 0.403, respectively).

During the follow-up period, local recurrence occurred in 2 of 59 lesions with incomplete resection. One recurrence occurred 29 months after ESD for hybrid GED, and the other, 55 months after ESD for adenomatous GED. The morphologic type of both recurrent lesions was adenomatous. In these 2 lesions, additional endoscopic treatment (argon plasma coagulation) was performed, and no further recurrence was noted during the follow-up period.

### Incidence of gastric cancer in patients with GED

Of 149 patients with GEDs, gastric cancer was detected in 21 (14.1%) patients, of whom 13 and 8 had synchronous and metachronous cancer, respectively. All gastric cancers were endoscopically diagnosed as EGC. Gastric cancer was detected in 11 (16.2%), 5 (10.9%), and 5 (14.3%) patients with adenomatous, foveolar, and hybrid type lesions, respectively. (*p* = 0.726).

### Clinicopathologic similarities of GEDs in patients with multiple GEDs

The clinicopathologic characteristics of patients with multiple GEDs (n = 40) are shown in Table 4. Approximately two-thirds of the lesions showed similarities in terms of the morphologic type, location, and macroscopic shape compared to the primary lesions.

**Table 4:** **Clinicopathologic characteristics of lesions in patients with multiple gastric epithelial dysplasias**

Primary → Secondary lesion	Synchronous GED	Metachronous GED	Total
	(n = 18)	(n = 22)	(n = 40)
GED type			
A → A/F/H	8/2/1	9/0/1	17/2/2
F → A/F/H	1/3/0	4/1/0	5/4/0
H → A/F/H	1/1/1	3/1/3	4/2/4
Same type	12 (66.7)	13 (59.1)	25 (62.5)
Location			
Body → Body/Antrum	3/4	6/5	9/9
Antrum → Body/Antrum	5/6	3/8	8/14
Same location	9 (50.0)	14 (63.6)	23 (57.5)
Macroscopic shape			
I&IIa → I&IIa/IIb&IIc	9/5	7/8	16/13
IIb&IIc → I&IIa/IIb&IIc	2/2	2/5	4/7
Same shape	11 (61.1)	12 (54.5)	23 (57.5)

Values are expressed as n (%).

GED, gastric epithelial dysplasia; A, adenomatous type; F, foveolar type; H, hybrid type.

## Discussion

The increased use of esophagogastroduodenoscopy recently has resulted in an increase in the diagnosis of GED and the subsequent endoscopic treatments of this lesion. Although several studies have previously reported on the clinical and endoscopic characteristics according to the morphologic types [15-19], numerous questions still remain concerning the clinical significance of each type of GEDs, including the long-term follow-up outcomes. In addition, these previous studies included only relatively small numbers of cases. In the present study, we compared the clinicopathologic characteristics of GEDs according to the morphologic types and evaluated the long-term follow-up outcomes of each type (synchronous and metachronous lesions).

In the present study, we found that GED was more prevalent in men (male:female ratio, 2.2:1), which is similar to the results of previous studies [6,7]. However, there was no difference in the male:female ratio according to the morphologic types.

The natural history of *H. pylori* infection in the stomach is to go through a cascade of events, including non-atrophic gastritis, atrophic gastritis, intestinal metaplasia, dysplasia, and finally cancer [28,29]; and the presence of *H. pylori* infection has showed to be associated with an increased risk of progression to dysplasia or gastric cancer, with an odds ratio of 1.8 [30]. In the present study, the frequency of *H. pylori* infection was 79.6% in patients with GED, which is substantially higher than the frequency reported in the general population (59.6%) [31,32]. However, there was no difference in the frequency of *H. pylori* infection according to the morphologic types. Although this was not an epidemiological study, our results support the close relationships between *H. pylori* infection and GED, irrespective of morphologic types.

It has been well established that GEDs occur throughout the stomach, with a slight antral predominance, and that they can range in size from a few millimeters to several centimeters [33-35], which is similar to our results (antrum:body ratio, 1.4:1). However, in this study, we moreover demonstrated that GEDs show distinct endoscopic and histopathologic features according to the morphologic types. We found that adenomatous GEDs were more likely to occur in the gastric body and lesser curvature side of the stomach, whereas foveolar GEDs were mainly located in the gastric antrum and non-lesser curvature side of the stomach. In addition, foveolar GEDs were smaller, and were normal/red colored and showed a flat/depressed shape more frequently than adenomatous GEDs. From a histologic viewpoint, foveolar GEDs were more likely to show high-grade dysplasia than were adenomatous GEDs, which is consistent with the results of our previous study [19]. An association between macroscopic shape and histologic grade has been suggested in a previous study [36], with GEDs with a depressed shape showing higher proportions of reddish color and severe atypia or carcinoma compare to GEDs with non-depressed shapes. These findings concur with our findings that foveolar GEDs predominantly showed normal/reddish color, flat/depressed shape, and high-grade morphology. Furthermore, in the present study, of the 8 metachronous gastric cancers detected during the follow-up period, only one case was a signet ring cell carcinoma, and this occurred in a patient with a foveolar GED, which is consistent with the results of previous studies suggesting that the foveolar type is associated with high-grade histologic features and poorly-differentiated adenocarcinomas [3,19,22,23]. We speculate that these differences observed between GED types might reflect differences in the tumorigenesis of each GED type. However, further large-scale studies are required to clarify this.

In the present study, the complete resection rate in adenomatous type lesions was 78.4%, which was significantly lower than in foveolar and hybrid type lesions (84.5% and 92.0%, respectively). We hypothesize that the cause for this phenomenon may be because most GEDs with adenomatous type were low-grade and considered more discrete lesions owing to their whitish color and elevated/protruded morphology compared to those of the other 2 types, and that, consequently the endoscopists may have tended to perform endoscopic resection less carefully, such as marking closely around the lesion.

Treatment strategies for incomplete resection after endoscopic resection of GEDs have not been well established. Considering the indolent behavior of GED, we performed annual follow-up for incompletely resected GEDs. During the follow-up period, local recurrence occurred in only 2 of 59 lesions with incomplete resection. In these 2 lesions, additional argon plasma coagulation was performed, after which further recurrence did not occur. Local recurrence is generally caused by small remnants being left on the margins after endoscopic resection. However, in the present study, the recurrence rate in cases with incomplete resection was very low (3.4%). The reason for this might be the uncertainty of judging a cut margin because of the burning effect of electrosurgical devices on the residual tumor cells in positive margins. Our results suggest that close endoscopic surveillance without additional treatment may be an acceptable option for GEDs with incomplete resection.

A major concern in the management of GEDs is the inability to predict in which patient cancer will occur. Because it is often difficult to distinguish between the histologic grades from endoscopic findings, and because there are commonly discrepancies between the pre-endoscopic resection and post-endoscopic resection diagnoses [37-39], treatment modalities for GEDs ensuring accurate diagnosis and potentially curative resection, such as ESD or EMR, are needed. However, ESD or EMR can only remove gastric neoplasms in the part of the gastric mucosa containing the visible lesion with minimal surrounding normal tissue. Therefore, there is always a risk of occurrence of synchronous or metachronous gastric neoplasms in other sites, as GEDs are known to be indicators of an increased risk of synchronous adenocarcinoma elsewhere in the stomach [12,40].

One or more primary carcinomas can coexist at the time of diagnosis (synchronous), or develop consequently (metachronous), sometimes years after resection of the first GEDs. In the present study, gastric cancers were found on 21 out of 149 patients with available long-term follow-up information (14.1%). This rate is very high compared to the incidence of gastric cancer in the general population (0.3%) and individuals participating in health-screening programs (0.44%) [41,42]. Conversely, there were no cases of tumor-related deaths due to synchronous or metachronous cancer in this study. The prognosis in patients with gastric cancer depends largely on the stage at diagnosis [43], and in the present study, the median time interval from endoscopic resection of GED to metachronous cancer was approximately 34 months, and all synchronous and metachronous cancers were found at an early stage. Although the purpose of this study is to evaluate differences in the follow-up according to the three histologic types, we added an analysis based on grading of the lesions (low- and high-grade). There were no statistically significant differences in the incidence of synchronous and metachronous lesions. Despite numerous reports on the characteristics of synchronous and metachronous lesions after endoscopic treatment, the appropriate surveillance strategies after gastric endoscopic treatment remain unclear. On the basis of our results, we believe that the meticulous endoscopic follow-up in patients with GEDs after endoscopic resection may lead to the diagnosis of gastric cancer at an early stage, thus improving the survival rate, and regular endoscopic examinations and histologic controls (for example annually) are advisable.

Furthermore, about two-thirds of synchronous and metachronous GEDs were found at similar locations as the primary lesion in the present study. Hence, careful observation around the site of the primary lesion may be helpful for detecting synchronous or metachronous lesions and to reduce the risk of overlooking these lesions during follow-up endoscopy.

To our knowledge, this is the largest study to show morphologic characterization of GEDs to date, and the first study to reveal the long-term outcomes of GEDs after endoscopic resection according to morphologic types. However, our study has some limitations. First, there may have been a potential selection bias resulting from the retrospective nature of this single-center study. In our study, many patients were not included in the analysis of long-term outcomes because they did not undergo endoscopy at our hospital and hence their results were not available. The reasons for loss to follow-up in this study could be explained by the fact that after endoscopic treatment of GEDs, some patients choose to undergo follow-up endoscopy at other hospitals for various reasons such as the cost of endoscopy, the distance to the hospital, personal reasons, or the National Cancer Screening Program. In Korea, the National Cancer Screening Program provides endoscopy free-of-charge every 2 years for individuals older than 40 years. If the data for the patients lost to follow-up within 12 months of ESD had been included in our analysis, the study may have shown different results. Second, the follow-up period (median 37.3 months) was relatively short for a study of long-term results, and further large-scale prospective studies involving a greater number of patients and longer follow-up periods will be needed to obtain more exact information on the clinicopathologic features of each subtype of GEDs and the long–term outcomes of GEDs after endoscopic resection. Third, we classified GEDs as adenomatous, foveolar, or hybrid types according to their morphologic features only; rthese types were determined by a single expert gastrointestinal pathologist. However, histologic evaluation alone, even if performed by an expert pathologist, may fail to evaluate the morphologic type correctly. Therefore, it could have been more accurate and more objective if at least two expert pathologists had analyzed the morphologic type. Furthermore, in such situations, mucin expression patterns as determined by immunohistochemistry are helpful to obtain detailed information on the differentiation of GEDs. Although we did not evaluate the mucin expression patterns in the present study, we previously found that foveolar and hybrid types are more often positive for MUC5AC, whereas the adenomatous type is more often positive for CD10 [19]. Since there results have already been reported previously, we did not perform immunohistochemical analysis to investigate the concordance of histologic and immunohistochemical classification of GEDs in the present study.

## Conclusions

Our study demonstrated the clinicopathologic characteristics of GEDs and long-term follow-up results after endoscopic resection according to morphologic categorization. Foveolar type lesions were found to have a depressed shape, smaller size, normal/reddish color, and antral predominance and to be high-grade lesions significantly more frequently than adenomatous type lesions. Irrespective of the morphologic type, synchronous and metachronous lesions were commonly found after endoscopic resection of GEDs. Therefore, close follow-up surveillance after endoscopic resection of GEDs should be performed for all patients, and endoscopists should make note of similarities among multiple lesions.

## Abbreviations

GED: Gastric epithelial dysplasia
ESD: Endoscopic submucosal dissection
EMR: Endoscopic mucosal resection
EGC: Early gastric cancer
